# Outcome Measures for Evaluating the Effect of a Multidisciplinary Intervention on Axial Symptoms of Parkinson's Disease

**DOI:** 10.3389/fneur.2020.00328

**Published:** 2020-05-12

**Authors:** Raquel Bouça-Machado, Filipa Pona-Ferreira, Nilza Gonçalves, Mariana Leitão, Ricardo Cacho, Ana Castro-Caldas, Joaquim J. Ferreira, and CNS Multidisciplinary Team

**Affiliations:** ^1^Instituto de Medicina Molecular, Lisbon, Portugal; ^2^CNS—Campus Neurológico Sénior, Torres Vedras, Portugal; ^3^Laboratory of Clinical Pharmacology and Therapeutics, Faculdade de Medicina, Universidade de Lisboa, Lisbon, Portugal

**Keywords:** parkinson's disease, axial symptoms, multidisciplinary, outcome measure, efficacy, sample size

## Abstract

**Introduction:** The satisfactory symptomatic control of the axial symptoms of Parkinson's disease (PD) remains challenging. As these symptoms are an important cause of disability, new therapeutic strategies should be developed and evaluated. To do this, it is necessary to select the outcomes to be measured and reported in a clinical trial. In this study, we sought to identify the most responsive outcome measures for assessing the efficacy of a multidisciplinary intervention on the axial symptoms of PD.

**Methods:** An exploratory prospective clinical study was conducted. PD patients engaged in a pre-defined multidisciplinary intervention program for parkinsonian patients were assessed at admission and discharge by a multidisciplinary team. The responsiveness to intervention was evaluated and the smallest sample size needed to enable statistically significant results for an expected 30% change from baseline for each outcome was calculated.

**Results:** Twenty-two patients were included in the study. The effect size detected varied between 0.04 and 0.83. The Movement Disorder Society—Unified Parkinson's Disease Rating Scale (MDS-UPDRS) total score and each subsection, the N-FOG questionnaire, the 10-m walk test, and Frenchay Dysarthria Assessment-2 Edition (FDA-2) showed a medium to large effect size. Sample size calculations for 90% power and assuming 30% change from baseline ranged from eight to 180 participants. The outcome measures that require a small number of participants to enable statistically significant results were the FDA-2 rating scale (*n* = 4 participants), the MDS-UPDRS total score (*n* = 9), the 10-m walk test (*n* = 9), and the MDS-UPDRS motor examination (*n* = 10).

**Conclusions:** The MDS-UPDRS part III and total score and the 10-m walk test were the outcomes with the best responsiveness to a multidisciplinary intervention and required a small number of participants to enable statistically significant results. Further studies are needed to clarify the suitability of the Timed Up and Go test.

## Introduction

Axial symptoms associated with Parkinson's disease (PD) have an important impact on the patients' quality of life and are a risk factor for institutionalization and death (1, 2). They comprise a set of signs, including cognitive, speech, swallowing, and urinary symptoms, associated with posture deformations, posture instability, and gait disorders (1–3). Axial symptoms increase in frequency and severity throughout disease progression. There is presently no satisfactory pharmacological intervention for their management (1–3).

Currently, PD management follows, in the majority of cases, a “monodisciplinary” approach focused on the use of pharmacological interventions (3). To optimize patient care, a comprehensive multidisciplinary approach that takes into account the complexity and diversity of PD symptoms appears preferable (3, 4). However, there are no data from large clinical trials to support this approach (1, 3).

The selection of outcomes to be measured and reported in a clinical trial is crucial (5). This should reflect the main goal of the trial and has also implications for sample size calculation (5).

Responsiveness to change is a clinimetric property of measurement instruments that is characterized by the ability of an outcome measure to accurately detect meaningful changes in clinical state or health over time (5, 6). This is critical for establishing the smallest clinically significant change in the measurement (minimal clinically important difference) and the smallest sample size needed to enable statistically significant results, thus defining the magnitude of the effort needed (5, 6).

We sought to identify which previously validated outcome measures used for assessing the different axial symptoms of PD are the most responsive for assessing the efficacy of a multidisciplinary intervention on these symptoms.

## Methods

### Study Design

An exploratory prospective clinical study was conducted.

### Objective

We aimed to study the most responsive outcome measures for assessing the efficacy of a multidisciplinary intervention on the axial symptoms of PD.

### Participants

The study participants were recruited from the Campus Neurológico Sénior (CNS), a tertiary specialized movement disorders center in Portugal. Patients were eligible if they had a diagnosis of PD according to the International Parkinson and Movement Disorder Society criteria, had engaged in a multidisciplinary therapeutic program for parkinsonian patients in CNS between January and September 2019, and if they agreed to participate. The exclusion criteria were the inability to adopt a standing position and/or to walk 3 m, postural instability compromising patient safety during the assessment, and the presence of cognitive defects that prevent understanding of the test instructions. The study was undertaken with the understanding and the written consent of each participant, with approval from the CNS Ethics Committee (Ref. 10/19) and in compliance with national legislation and the Declaration of Helsinki. The subjects were required to agree to all aspects of the study and were able to leave the study at any time.

### Therapeutic Intervention

**Figure 1:**
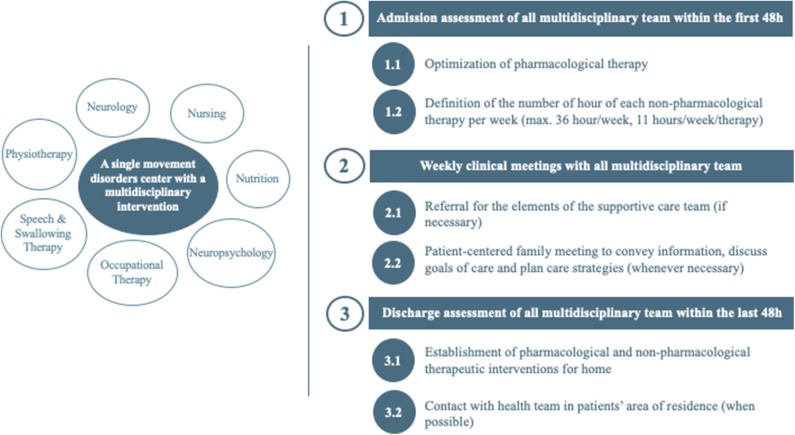
Multidisciplinary team and intervention organizational structure.

The multidisciplinary intervention combined pharmacological and non-pharmacological therapies in different intensity, complexity, and frequency in the gym, pool, and/or outdoor spaces, according to the patient's needs, as identified in the admission assessment. The patients were assessed in the first 48 h after admission by each health professional (Figure 1). The neurologists and the nurses collected demographical and clinical data, past medical history, and current pharmacological therapeutic interventions. Following the admission evaluations, the multidisciplinary team defined the therapeutic program, including the number of sessions that each participant would have with each specialty.

The participants received a mean number of 20 h/week of individually tailored neurorehabilitation sessions. These included:

- Physiotherapy sessions—intensive programs of physical capacity training, gait, mobility, balance, sensorimotor coordination, and development as well as teaching the patient and usual caregivers of movement facilitating or adaptive strategies to enhance functionality;
- Speech and swallowing therapy—stimulation and training of speech intensive programs, language, and communication in order to increase speech intelligibility and stimulate self-communicative skills as well as performing teaching and training of usual caregivers in order to enhance the patient's communicative effectiveness;
- Occupational therapy—assessment strategies and treatment of occupational performance problems, which aim to maximize the users' balance and adaptation to their daily skills and tasks, supported by specific methodologies and activities;
- Neuropsychology—cognitive stimulation programs aimed at enhancing the cognitive (e.g., memory and attention) and social functioning of each patient according to the characteristics of each specific situation and to the predominant difficulties.

Throughout the implementation of the therapeutic program, the neurology, nursing, and nutritionist professionals provided support.

A multidisciplinary clinical meeting was performed twice weekly to discuss the case of each patient. The need for adjustment of the pharmacological and the non-pharmacological therapeutic interventions, the need for a meeting with family or to contact community resources, and other relevant issues for the patients' care were reviewed.

### Clinical Assessment Protocol

The patients were assessed at admission and prior to discharge. Data on the following parameters were collected:

- Disease severity: Movement Disorder Society—Unified Parkinson's Disease Rating Scale (MDS-UPDRS) total score and score from each sub-section (7), Hoehn and Yard scale (7, 8), and Clinical and Patient Global Impression of Severity and Change (9);
- Speech and swallowing function: Voice Handicap Index (10), Frenchay Dysarthria Assessment-2 Edition (FDA-2) (11), and Swallowing Clinical Assessment Score in PD (12);
- Motor function: The Timed Up and Go (TUG) test with and without a cognitive dual-task (13–15), 10-m walk test (15–18), Mini-Best test (15, 19, 20), and the New Freezing of Gait Questionnaire (21, 22).

Based on previously published studies (23, 24), the MDS-UPDRS-III sub-items for speech (item 3.1), neck rigidity (item 3.3), posture (item 3.13), gait (item 3.10), freezing of gait (item 3.11), arising from chair (item 3.9), postural instability (item 3.12), and total axial signs (sum of items: 3.1, 3.10–3.12) were studied separately as a composite score for the assessment of PD axial symptoms.

### Statistical Analysis

Descriptive statistics were used for demographic, clinical, and therapeutic data.

Continuous outcomes were defined as the total change from baseline for all the previously mentioned outcome measures and presented as a mean ± standard deviation (SD). Wilcoxon test was applied for each parameter to investigate the existence of a statistically significant difference between admission and at the end of the program; significance was achieved with a *p* <0.05. The responsiveness to change of each outcome measure was based on the calculation of the standardized mean effect size using Cohen's *d*. For Cohen's *d*, a value from 0.20 to 0.49 represents a small effect size, from 0.50 to 0.80 represents a medium effect size, and >0.80 represents a large effect size (25). To power analysis and sample size calculations, G^*^Power software was used. For power analysis, a significance level of α = 0.05 and a power = 1 –β = 0.90 were assumed. To explore the variability of the included measurement instruments, a power analysis assuming 30% of change from baseline and using the mean SD of change from baseline was calculated for each instrument. Based on this information, the outcome measures requiring a smaller sample, if used as primary outcomes of clinical trials, were identified. The choice of the magnitude of effect (30%) was based on the minimal clinical difference reported for the Timed Up and Go test, a measurement tool recommended for assessing functional mobility, which is an outcome closely related with axial symptoms.

## Results

### Cohort General Data

Of the 54 PD patients who engaged in a CNS rehabilitation program between January and September 2019, a total of 22 were included in this study. The reasons for exclusion were lack of collaboration/missing data (29.6%, *n* = 16), motor inability to perform the assessments (18.5%, *n* = 10), and cognitive impairment and behavioral disturbances (9.3%, *n* = 5). The mean age was 73.6 ± 6.9 years, and the number of male patients was 15 (71.4%). The average disease duration since diagnosis was 7.4 ± 4.9 years, with a mean Hoehn and Yahr stage of 2.9 ± 0.8 at admission. The mean clinical and the patients' global impression of change was 2.6 ± 1.0 (much improved, *n* = 20) and 2.6 ± 0.8 (much improved, *n* = 16), respectively (Supplementary Table 1). The patients' demographics and clinical characteristics at baseline are summarized in Table 1. Tables 2, 3 report the data on statistical significance, responsiveness to change, and power analysis of each measurement tool.

**Table 1:** Demographic and clinical data.

**Demographic features (*****n*** **=** **22)**				
Age [Mean (SD)]				73.6 (± 6.9)
Time since diagnosis [Mean (SD)]				7.4 (± 4.9)
Male sex [% (*n*)]				71.4% (15)
Tremor—First symptom [*n* = 13; % (*n*)]				38.5% (5)
Hours of physical activity/week (Median [Min, Max], *n* = 22)				2.5 [0,11]
**Clinical data (mean (SD), [range])**				
	**Admission**	**Discharge**	**Change**	**MCID**
MDS-UPDRS I (range, 0–52; *n* = 22)	14.6 (± 5.6)	10.0 (± 5.4)	−4.5 (± 5.8), [−17, 5]	−2.64
MDS-UPDRS II (range, 0–52; *n* = 22)	21.1 (± 9.2)	15.3 (± 9.3)	−5.7 (± 7.3), [−27, 4]	−3.05
MDS-UPDRS III (range, 0–132; *n* = 22)	45.0 (± 14.7)	37.9 (± 13.9)	−7.1 (± 11.0), [−6, 17]	−3.5
MDS-UPDRS IV (range, 0–24; *n* = 22)	2.3 (± 3.9)	2.0 (± 2.7)	−0.3 (± 2.6), [−5, 7]	Unk
MDS-UPDRS Total (range, 0–260; *n* = 22)	82.6 (± 23.8)	65.2 (± 24.6)	−17.4 (±1 9.0), [−12, 13]	−7.1
Hoehn and Yahr stage (range, 1–5; *n* = 22)	2.9 (± 0.8)	3.0 (± 0.8)	0.1(± 0.4), [−1, 1]	NA
		**Severity**		**Change**
Clinical Global Impression (*n* = 16)		4.1 (± 1.6)		2.6 (± 1.0)
Patient Global Impression (*n* = 20)		4.3 (± 1.0)		2.6 (± 0.8)

**Table 2:** Multidisciplinary intervention and axial symptoms assessment.

**Multidisciplinary intervention [Mean (SD)**, ***n*** **=** **22]**			
Duration of the program (weeks)		3.0 (± 2.0)	
Levodopa equivalent daily doses (LEDD)	**Admission**		**Discharge**
	1023.1 (± 835.1)		1040.3 (± 689.2)
Physiotherapy session/week		11.0 (± 1.0)	
Speech and swallowing therapy session/week		6.0 (± 2.0)	
Neuropsychology sessions/week		4.0 (± 1.0)	
**Axial symptoms assessment (mean (SD), [range])**			
	**Admission**	**Discharge**	**Change**
**MDS-UPDRS—Axial symptoms items score** (range, 0–68)	21.7 (±7.6)	15.7 (±8.4)	−6.0 (±7.9), [−11, 9]
**Speech and swallowing function**			
Voice handicap index (*n* = 14)	35.2 (± 30.2)	37.4 (± 26.0)	4.2 (± 26.2), [−64, 53]
FDA-2 (*n* = 17)	76.4 (± 12.5)	82.8 (± 11.3)	6.4 (± 8.9), [−7.5, 23.5]
SCAS-PD (*n* = 14)	29.8 (± 33.4)	20.6 (± 25.0)	−9.2 (± 14.6), [−35, 18]
**Motor function**			
Timed up and go (*n* = 22)	23.9 (± 31.2)	16.3 (± 13.9)	−7.7 (± 18.5), [−81.4, 4.0]
Timed up and go with cognitive DT (*n* = 20)	31.8 (± 58.0)	19.1 (± 19.9)	−12.7 (± 39.5), [−173.2, 8.7]
**10-meter walk test**			
Velocity (*n* = 22)	0.8 (± 0.3)	1.0 (± 0.3)	0.2 (± 0.3), [−0.3, 0.8]
Number of steps (*n* = 22)	22.8 (± 7.2)	19.4 (± 5.2)	−3.4 (± 5.5), [−17, 4]
Mini-best test (*n* = 22)	15.1 (± 8.2)	17.7 (± 6.3)	2.6 (± 6.6), [−6, 20]
New freezing of gait questionnaire (*n* = 14)	9.2 (± 9.3)	4.4 (± 8.4)	−4.9 (± 7.7), [−23, 0]

**Table 3:** Outcome measures responsiveness to change.

	**Outcome measures responsiveness to change**			
	***p***	**Cohen's d**	**Sample size (90% Power, real change)**	**Sample size (90% Power, 30% change from baseline)**
**Disease Severity**				
MDS-UPDRS I	0.002	0.83	18	22
MDS-UPDRS II	0.001	0.62	30	17
MDS-UPDRS III	0.014	0.49	45	10
MDS-UPDRS IV	0.474	0.09	1,400	147
MDS-UPDRS Total	0.001	0.72	23	9
MDS-UPDRS—Axial symptoms items score	0.499	−0.04	9,730	35
Hoehn and Yahr stage	0.317	−0.11	675	5
**Speech and swallowing function**				
Voice Handicap Index (*n* = 14)	0.778	−0.08	1,745	75
FDA-2 (*n* = 17)	0.014	−0.54	39	4
SCAS-PD (*n* = 14)	0.050	0.31	115	31
**Motor function**				
Timed Up and Go (*n* = 22)	0.039	0.32	136	74
Timed Up and Go with Cognitive DT (*n* = 20)	0.191	0.29	172	180
10-meter walk test (velocity)	0.004	0.54	40	9
Mini-Best test (*n* = 22)	0.120	−0.35	88	25
New Freezing of gait Questionnaire (*n* = 14)	1.000	0.55	38	88

### Responsiveness to Intervention

A medium to large effect size (*d* = 0.50–0.80) was found in the MDS-UPDRS part I (*d* = 0.83), II (*d* = 0.62), III (*d* = 0.49), and total (*d* = 0.72), in the N-FOG questionnaire (*d* = 0.55), in the 10-m walk test (*d* = 0.54), and in the FDA-2 (*d* = −0.54).

### Sample Calculation

Assuming a 30% change from baseline, the outcome measures that require a small number of participants to enable statistically significant results were the FDA-2 rating scale (*n* = 4 participants), the MDS-UPDRS total score (*n* = 9), the 10-m walk test (*n* = 9), and the MDS-UPDRS motor examination (*n* = 10).

Using the real change from baseline, the outcome measures that require a small number of participants to enable statistically significant results were the MDS-UPDRS part I (*n* = 18), the MDS-UPDRS total score (*n* = 23), the N-FOG questionnaire (*n* = 38), the FDA-2 rating scale (*n* = 39 participants), the MDS-UPDRS motor examination (*n* = 45), and the 10-m walk test (*n* = 40).

## Discussion

From the set of outcome measures used to evaluate the efficacy of the multidisciplinary program for the axial symptoms associated with PD, four were able to detect a medium to large effect size (*d* ≥ 0.5).

According to our results and focusing on the responsiveness to change and the sample needed to ensure 90% power, the most attractive outcome measures seem to be the MDS-UPDRS total score and parts I, II, and III, the FDA-2, and the 10-m walk test.

### The Movement Disorder Society—Unified Parkinson's Disease Rating Scale

The MDS-UPDRS was the outcome measure with the best responsiveness to change to the multidisciplinary intervention and the measure that required the smallest sample size if used as a primary outcome. The total score of the scale offers a subjective and an objective assessment of the axial symptoms of PD (26). Although very complete and considered as the gold standard for testing the efficacy of a particular intervention over a comparator in PD, this is a very time-consuming rating scale that cannot always be used in everyday clinical practice. Several studies have sought to overcome this limitation by only using the motor examination subsection of the scale instead of the total score.

Although based on the MDS-UPDRS part III, the responsiveness to change and the power analysis results of the MDS-UPDRS axial symptoms items score (23, 24) were very different (*d* = −0.04, 9,730 participants for 90% power). We believe that these results can be partly explained by the heterogeneity and the small sample size. However, comparing the results of this score with the other outcome measures used to assess the same constructs suggests that this would not be the most sensitive outcome for measuring efficacy in a future similar study.

### The Frenchay Dysarthria Assessment

The FDA-2 reached a medium effect size (*d* = −0.54), requiring only 39 participants to obtain 90% power using this magnitude of effect (four participants if using 30% change from baseline). These results are very interesting. However, the FDA-2 is only focused on speech and swallowing problems; it might not be the most illustrative outcome measure for assessing the axial symptoms of PD (11). Nonetheless, it would be interesting to study its correlation with the MDS-UPDRS, the Patient Global Impression, and the Clinical Global Impression scores.

### The 10-m Walk Test

After the MDS-UPDRS part III, the 10-m walk test, which focuses on motor assessment, was found to be the measure most sensitive to the intervention (*d* = 0.54) and had a more interesting power analysis result than the former (40 participants, or nine if using 30% change from baseline). Despite the good results and its ease of use, this clinical test only focuses on gait velocity, with low to medium correlation with balance tests and other measures of disease severity. For this reason, this test may not be representative enough of axial symptoms to be used as the primary outcome in future trials (15).

### The Timed Up and Go Test

Although the TUG test did not have the best results concerning responsiveness to change and power analysis, we believe that this outcome measure merits discussion.

The TUG test has been validated and recommended as a measurement tool for assessing functional mobility in PD, including the assessment of gait, transfers, and turns (15, 27).

Functional mobility is described as a person's physiological ability to move independently and safely in a variety of environments in order to accomplish functional activities or tasks and to participate in the activities of daily living at home, at work, and in the community (27). In PD, functional mobility limitations have a multifactorial nature, including the presence of rigidity, postural deformities (e.g., camptocormia or antecollis), and deficits in gait, balance, and transitions, which are all aggravated in the presence of cognitive impairments, orthostatic hypotension symptoms, and fatigue complaints (27).

The TUG test gives a numerical value to the global concept of functional mobility, allowing the objective quantification of deficits and change over time (27). As functional mobility is so dependent on axial symptoms, this can be a particularly useful tool for assessment and monitoring over time and for determining the efficacy of therapeutic interventions. While the sample size limitation may underestimate sensitivity, we believe that the TUG test should be assessed in a larger study as a potentially interesting primary outcome for assessing a multidisciplinary intervention in PD.

### Effectiveness of Multidisciplinary Interventions for the Axial Symptoms Associated With PD

Currently, while there are high-quality research programs studying the pharmacological treatment of the different axial symptoms in PD, there continues to be a lack of evidence on the efficacy of multidisciplinary interventions (1). Axial symptoms respond poorly to dopamine replacement therapy, and their satisfactory symptomatic control remains challenging (2, 3). Given their notable clinical importance—reduced mobility, loss of independence, recurrent falls, and subsequent injuries—new methodologies need to be explored (2, 3, 28).

Although this was not a trial intended to study efficacy and we cannot make conclusions on this topic, a medium to large effect size was found on more than one measure, and patients and health professionals perceived a benefit. We believe that our data provide information about and reinforce the importance of exploring this potentially interesting path for improving the management of axial symptoms in PD. Our results are supported by a 2005 study (1) on the efficacy of a multidisciplinary treatment program on PD patients' long-term outcomes. The authors reported the positive effects of a multidisciplinary approach in improving and maintaining PD patients' axial motor symptoms.

### The Use of Technology-Based Outcome Measures in PD Axial Symptoms Assessment

In the last decades, technology-based objective measures (TOMs) have been increasingly used in the assessment of PD axial symptoms, with the added value of allowing for a continuous, more ecological, and accurate assessment of the patients' daily routine (29). In a complex and fluctuating disease like PD, this type of quantitative information is crucial. Although still very new and fragile, TOMs seem very promising as outcome tools to enable early diagnosis, a more efficient assessment of disease progression, and therapeutic interventions and to decrease the duration and burden of PD clinical trials (29, 30). However, the use of TOMs is still limited, among others, by algorithm development, and the definition of the parameters are clinically relevant parameters (31–33).

### Limitations

Due to its exploratory design, this study presents two major limitations: a small sample size (*n* = 22) and high heterogeneity in the included population. Some patients, due to an impaired cognitive capacity, were not able to perform some of the assessments based on the questionnaires. We believe that these aspects may overestimate the variability of the instruments, influencing our results of sample size calculation. We expect that future studies with a less heterogeneous population need a smaller sample size to reach 90% power. As an open non-controlled study with no follow-up assessments, we hypothesize that, in future larger and longer controlled trials, the detected effect size will be smaller. Because this was not an efficacy study and some of the included outcome measures proved sensitive to change, despite the limitations mentioned, we believe that our results are informative and important for the design of future studies on this topic. Multidisciplinary interventions usually apply to more complex and heterogeneous patients. The use of an explicit, pre-defined intervention protocol in a single tertiary care center was intended to decrease the variability associated with the type of intervention.

## Conclusion

The use of standardized outcome measures is important not only for summarizing and better interpreting research findings but also to avoid an unnecessary increase in the completion time, complexity, and financial expenses of trials. Our results suggest that the MDS-UPDRS motor examination section and total score and the 10-meter walk test are interesting outcomes that should be considered as primary outcomes in future trials that seek to evaluate the effect of multidisciplinary interventions on the axial symptoms of PD. We also propose the study of the suitability of the TUG test in a larger trial.

## Data Availability Statement

All datasets generated for this study are included in the article/Supplementary Material.

## Ethics Statement

The studies involving human participants were reviewed and approved by CNS Ethics Committee (Ref. 10/19), CNS—Campus Neurológico Sénior, Torres Vedras, Portugal. The patients/participants provided their written informed consent to participate in this study. Written informed consent was obtained from the individual(s) for the publication of any potentially identifiable images or data included in this article.

## Author Contributions

RB-M and JF contributed to the conception and design of the study. RB-M, FP-F, ML, RC, and AC-C performed the assessments. RB-M, NG, and JF performed the statistical analysis and drafted the manuscript. All the authors contributed to manuscript revision, read, and approved the submitted version.

## Conflict of Interest

The authors declare that the research was conducted in the absence of any commercial or financial relationships that could be construed as a potential conflict of interest.

## Supplementary Material

Supplementary Table 1 Individual patient data.
